# Repeated intra-articular injections of umbilical cord-derived mesenchymal stem cells for knee osteoarthritis: a phase I, single-arm study

**DOI:** 10.1186/s12891-023-06555-y

**Published:** 2023-06-13

**Authors:** Yunong Ao, Jiangjie Duan, Na Xiong, Nannan Qian, Rui Zhang, Liu Yang, Shicang Yu, Fuyou Wang

**Affiliations:** 1 Center for Joint Surgery, Southwest Hospital, Third Military Medical University (Army Medical University), Chongqing, China; 2 Department of Stem Cell and Regenerative Medicine, Institute of Pathology and Southwest Cancer Center, Southwest Hospital, Third Military Medical University (Army Medical University, Chongqing, 400038 China

**Keywords:** Osteoarthritis, Stem cell, Knee, Cartilage

## Abstract

**Introduction:**

Stem cell therapy has emerged as an effective treatment for multiple diseases, and some studies also demonstrate that it may be a promising treatment for osteoarthritis (OA). However, few studies have clarified the safety of repeated intra-articular injection of human umbilical cord-derived mesenchymal stem cells (UC-MSCs). To promote its application in treating OA, we conducted an open-label trial to investigate the safety of repeated intra-articular injections of UC-MSCs.

**Methods:**

Fourteen patients with OA (Kellgrene-Lawrence grade 2 or 3) who received repeated intra-articular injections of UC-MSCs were evaluated in three months of follow-up. The primary outcomes were the adverse events, and the second outcomes included visual analog scale (VAS), Western Ontario and McMaster Universities Osteoarthritis Index (WOMAC), Magnetic Resonance Observation of Cartilage Repair Tissue (MOCART) scores and SF-12 quality of life score.

**Results:**

A total of 5 of 14 patients (35.7%) experienced transient adverse reactions, which resolved spontaneously. All patients showed some improvement in knee function limitation and pain after receiving stem cell therapy. VAS score 6.0 to 3.5, WOMAC score 26.0 to 8.5, MOCART score 42.0 to 58.0, SF-12 score 39.0 to 46.0.

**Conclusion:**

Repeated intra-articular injection of UC-MSCs demonstrates safety in treating OA and does not induce serious adverse events. This treatment may transiently improve symptoms in patients with knee OA and may be a potential therapeutic option for OA.

## Introduction

Osteoarthritis (OA) is a chronic disease characterized by degeneration and destruction of articular cartilage [1]. The incidence of OA is closely related to the increase of age, and the prevalence in females is significantly higher than that in males [2]. Studies have reported that in people over 60 years of age worldwide, the incidence is about 10% in men and 18% in women, and up to 80% of patients with OA have limited mobility [3]. With the accelerated increasing of the aging population, it will become the fourth most disabling disease and even be significantly positively associated with all-cause mortality. The risk factors of OA mainly include obesity, abnormal anatomy, and joint injury [4]. Recent epidemiological studies have found that socioeconomic status, environmental factors and lifestyles are also closely related to the incidence of OA [5]. Its clinical manifestations are mainly joint pain, swelling and limited mobility. Cartilage degeneration, subchondral osteosclerosis, progressive narrowing of the joint space, osteophyte formation and synovial inflammation are the main pathological features [6]. Meniscal degeneration, infrapatellar fat pad inflammation, and fibrosis are also present [7]. The pathogenesis of OA is complex, and it is generally believed that the imbalance of synthesis and degradation of articular chondrocytes, extracellular matrix, and subchondral bone may be the main pathological mechanism [8]. Because cartilage has a unique physiological structure of lacking neural and vascular tissue, its ability of self-repair is extremely limited [9]. Current approaches to retard OA progression include weight control, glucosamine, and nonsteroidal anti-inflammatory drugs [10]. However, these treatments can only transiently relieve OA symptoms and do not delay the progression of OA, and patients eventually must receive joint replacement. Therefore, exploring an effective treatment strategy for OA is a major challenge for clinicians.

In recent years, great research progress has been made in the application of cell therapy in the medical field. Among them, mesenchymal stem cell therapy for a variety of diseases has achieved good therapeutic results [11]. Mesenchymal stem cells (MSCs) are a kind of cells with self-renewal characteristics and multi-lineage differentiation potential, which have a wide range of sources and can be isolated from various tissues, including bone marrow, adipose or umbilical cord [12]. MSCs can secrete a great number of cytokines (CKs), chemokines and growth factors (GFs), and different mediators can promote MSCs to differentiate directionally into different types of cells, including bone and cartilage [13]. It has been reported that after local injection of MSCs, cytokines were released through the paracrine system to promote the regeneration of endogenous cartilage tissue, inhibited the apoptosis of chondrocytes, and facilitated the repair of cartilage [14]. At the same time, MSCs co-cultured with chondrocytes from OA patients revealed that MSCs produced a small amount of pro-inflammatory factors and chemokines, which could significantly reduce inflammatory factors secreted by chondrocytes, such as interleukin (IL)-6 and IL-8 [15].

Umbilical cord (UC)-MSCs are multifunctional adult stem cells derived from umbilical cord tissue, which have the advantages of high cell content, strong proliferation ability, low immunogenicity, and convenient sampling, and have broad clinical application prospects in tissue engineering [16]. In previous clinical studies, intra-articular injection of adipose-derived MSCs has achieved certain clinical efficacy in the treatment of knee OA, but UC-MSCs are rarely used. In this study, repeated intra-articular injection of UC-MSCs was conducted to analyze treatment-related adverse events (AEs), and provide the basis for subsequent stem cell therapy of OA.

## Materials and methods

### Ethics

This study protocol was registered at ClinicalTrials.gov (NCT05160831) and approved by the committee of Army Medical University (KY2021087). It was conducted in accordance with Good Clinical Practice guidelines and the Declaration of Helsinki. Each participant understood the procedures and precautions, and signed the informed consent form.

### Study objective and design

This is a prospective single-arm study in which the safety and efficacy of the intra-articular UC-MSCs for the patients suffering from knee OA is investigated. Each patient received four intra-articular injections of UC-MSCs once a week, and AEs were recorded after each injection. A 3-month follow-up was performed after the last injection to assess joint function and pain improvement.

### Preparation and characterization of UC-MSCs

The stem cells used in this study were derived from the UC obtained during caesarean section, and all donors signed informed consents and underwent comprehensive sociological and medical testing before caesarean section to ensure that the procedure was in accordance with medical and ethical norms. UCs were washed with normal saline and sectioned into 1–2 cm long segments, vessels were stripped, and Wharton’s jelly was separated. Under sterile conditions, Wharton’s jelly was minced and placed into cell culture flasks containing stem cell growth medium (Dakewe Biotech, China). After approximately 7 days in culture, stem cells crawled out from Wharton’s jelly and were extracted for subculture.

**Fig. 1:**
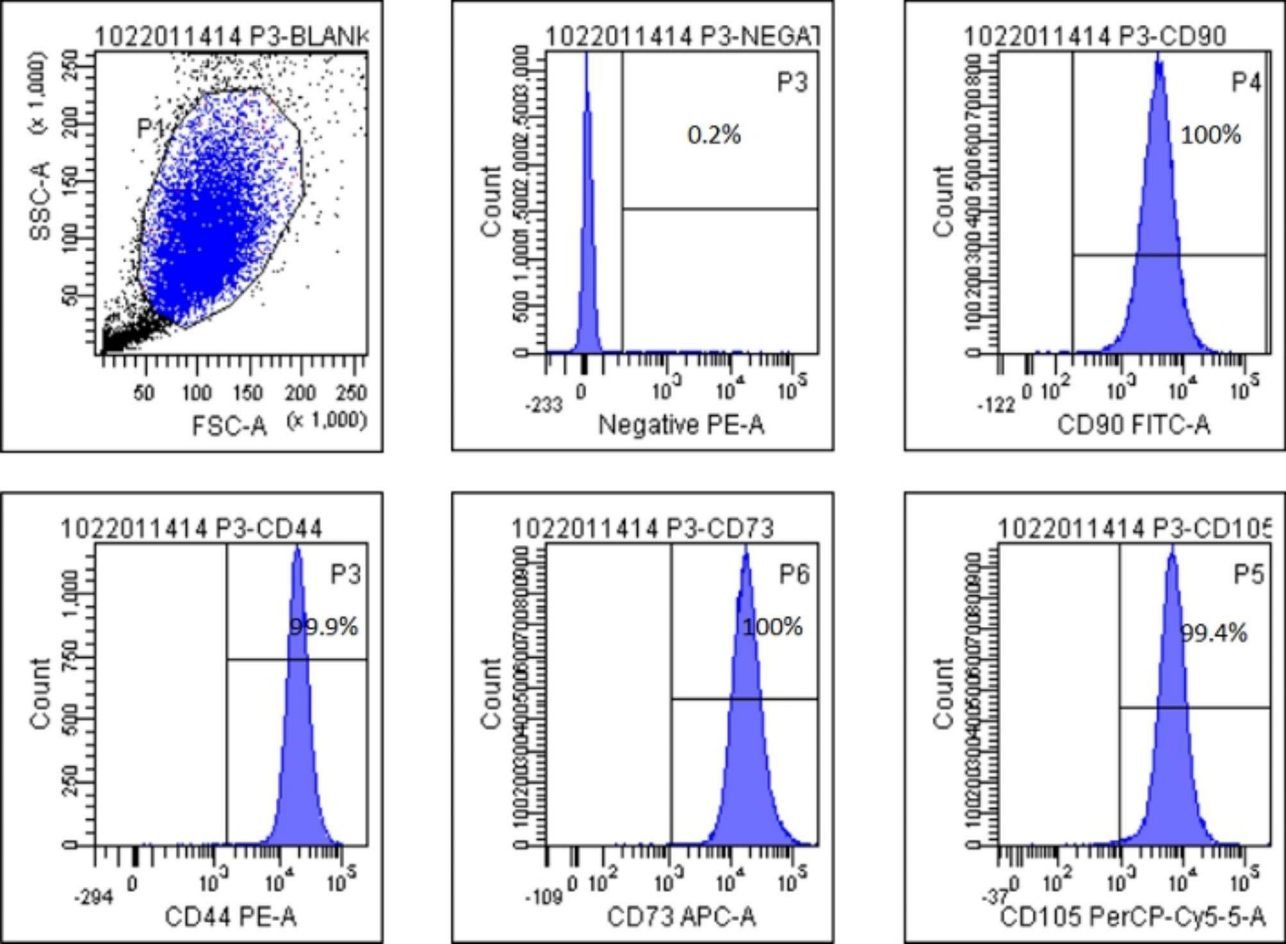
Characterization of UC-MSCs.

The cells were cultured with stem cell growth medium at 37 °C in a humidified atmosphere containing 5% CO_2_, wherein the culture medium was changed twice a week. UC-MSCs were cultured to passage 3 for pre-clinical testing, including the absence of macroscopic masses, bacteria, mycoplasma, syphilis, hepatitis B virus, hepatitis C virus, HIV, cytomegalovirus, and Epstein-Barr virus, and endotoxin (≤ 0.5 EU/mL). Flow cytometry (BD LSR Fortessa, USA) was used to identify stem cell phenotypes with antibodies (Thermo, USA). Briefly, cells cultured to P3 were centrifuged and resuspended, incubated with relevant antibodies, and subsequently detected using flow cytometry. Results for identity and purity were ≥ 95% positivity for CD73, CD90, CD44, and CD105, and negative expression for CD45, CD34, and CD14 (shown in Fig. 1). After ensuring that UC-MSCs compound was up to the standard for clinical application, cells were suspended in a volume of 3 mL saline with 1.5 × 10^7^ cells and loaded into 5 mL sterile syringe for subsequent injection.

### Participants and eligibility criteria

All fourteen patients enrolled in this study between December 2021 and June 2022 were diagnosed with knee OA by physical examination and radiographic evidence, and with grade 2 or 3 on the Kellgren–Lawrence scale [17]. In order to have a good consistency of the study and yield scientifically valuable results, strict inclusion and exclusion criteria were developed, as presented in Table 1.

**Table 1:** Eligibility criteria

Inclusion criteria	Exclusion criteria
1. Aged 18–70. 2. Diagnosed with grade 2 or 3 OA according to the Kellgren–Lawrence scale. 3. Ability to act autonomously. 4. Without mental illness. 5. No local or systemic infection. 6. No obvious contraindication for joint puncture. 7. Understand and agree to sign the informed consent form for the clinical trial protocol. 8. Comply with requirements of study visits during follow-up.	1. Body mass index (BMI)＞30 kg/m^2^. 2. Severe knee OA with severe varus and valgus deformity. 3. Pregnant or lactating women, or women who have a positive pregnancy test within 7 days prior to receiving study treatment. 4. Combined with severe cardiovascular, cerebrovascular, hepatic, and renal dysfunction. 5. Patients with immunodeficiency. 6. Being treated with immunosuppressive agents and corticosteroids. 7. Patients who are still participating in other clinical trials.

### Procedures

All intra-articular injections were performed by two experienced orthopedic surgeons in this study, choosing the lateral approach with knee extension. Each patient received intra-articular injection of UC-MSCs once a week for 4 times, and full-weight bearing was allowed after injection. Celecoxib was used to relieve pain for the first three days after treatment, 100 mg once daily. After the last injection, the clinical effects and AEs assessments were initiated by another orthopedic surgeon.

### Outcomes assessment

Western Ontario and McMaster Universities (WOMAC) index were applied to assess the knee function of patients [18]. Improvement in knee pain was evaluated using Visual Analog scale (VAS), and the quality of life was assessed by the Short-form 12 (SF-12) [19]. All patients underwent the above assessments before injection and at 12 weeks after the last injection. Magnetic resonance imaging (MRI) results were evaluated by a specialized radiologist according to the Magnetic Resonance Observation of Cartilage Repair Tissue (MOCART) score [20]. AEs were documented after each injection and presented in terms of incidence and relatedness with UC-MSCs intra-articular injection.

### Statistical analysis

Sample size in this study was not statistically determined, and the sample size of the study population was insufficient for conclusive statistical analysis. Descriptive data were presented as the mean (standard deviation [SD]), median (interquartile range [IQR]), and number (%).

## Results

### Patient demographics

A total of 14 patients were involved in this study, 4 males and 10 females, with a mean age of 58.29 ± 8.99 years (range, 46–70 years). After evaluation by orthopedic surgeon based on symptoms and radiographical outcomes, there were 8 people with K-L grade 2 and 6 people with grade 3. The mean BMI was 24.97 ± 2.69 (range, 19.61–28.89) kg/m^2^. All patients were followed for 3 months after receiving UC-MSCs intra-articular injection. Baseline characteristics of the patients was presented in Table 2.

**Table 2:** Baseline characteristics of the patients

Patients	Sex/Age	Body weight (kg)	Body mass index	Duration of disease (year)	Kellgren–Lawrence grade	Previous treatment
P1	F/53	57	22.27	4	2	TCM
P2	F/63	60	24.34	6	2	Acupuncture
P3	F/59	65	28.89	7	2	NSAIDs
P4	F/50	70	26.03	3	2	None
P5	M/68	66	25.78	9	2	Acupuncture
P6	F/70	63	28.76	11	3	Acupuncture
P7	F/62	60	24.97	6	2	Sodium hyaluronate
P8	M/46	69	26.29	3	2	TCM
P9	M/48	75	24.21	4	2	None
P10	F/67	65	25.39	12	3	Sodium hyaluronate
P11	F/47	52	20.83	1	2	None
P12	M/50	58	19.61	3	2	NSAIDs
P13	F/63	68	27.59	6	3	Acupuncture
P14	F/70	59	24.56	9	3	TCM, NSAIDs

TCM, Traditional Chinese medicine; NSAIDs, Non-steroidal anti-inflammatory agents

### Adverse events

The incidence of AEs was 35.7% (presented in Table 3), but the severity of AEs were all nonserious. Most of these AEs were associated with intra-articular injection, including joint pain, swelling, numbness and stiffness. Some other symptoms emerged during the first three days after injection, but all of them were transient and did not affect the patient’s normal activities. No postoperative complications associated with the intra-articular injection, such as injection site infection, were observed. All AEs recovered spontaneously after injection.

### Clinical outcomes

**Fig. 2:**
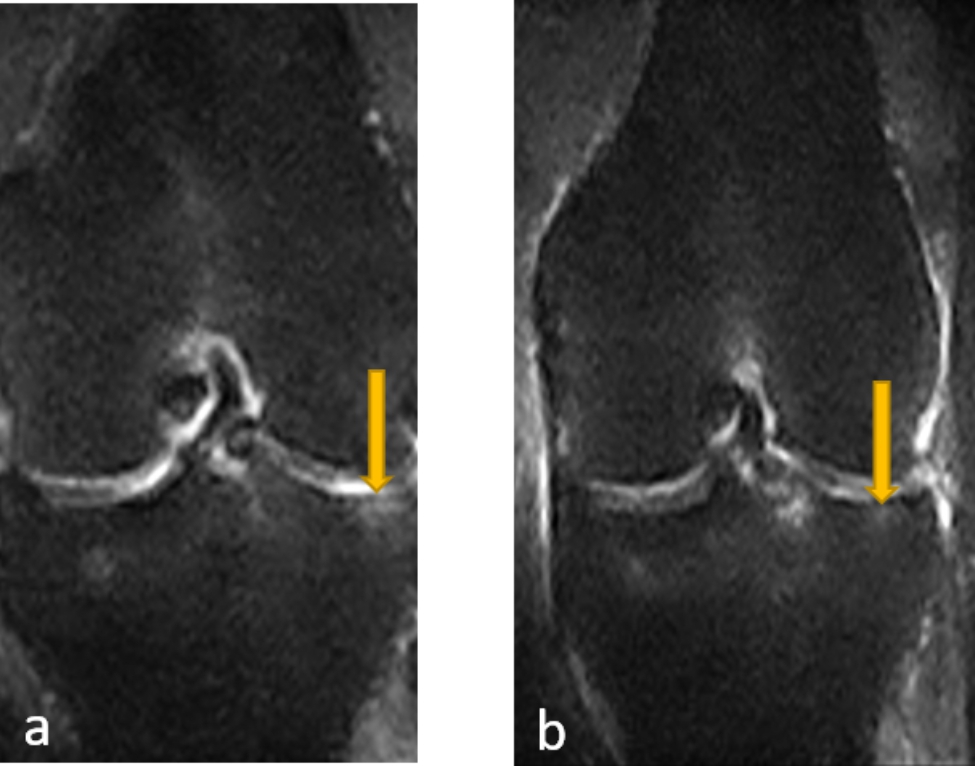
Representative MRI outcomes before injection **(a)** and 12 weeks after injection **(b)**, indicating subchondral bone edema improvement

Clinical outcomes were described in Table 4 for all evaluation index. VAS, WOMAC, SF-12 and MOCART scores at the final follow-up after injection improved compared with the preoperative scores, indicating that UC-MSCs intra-articular injection improved the function of knee and the quality of life. Representative MRI outcome was presented in Fig. 2.

**Table 3:** Adverse events

Case	Adverse events	Details	Severity	Outcome
1	Pain	Transient pain at the medial aspect of joint	Nonserious	Recovered
	Swelling	Transient swelling at the joint	Nonserious	Recovered
	Fever	Transient fever up to 37.6℃ at POD-2	Nonserious	Recovered
2	Pain	Transient pain at the lateral aspect of joint	Nonserious	Recovered
	Fever	Transient fever up to 37.5℃ at POD-1	Nonserious	Recovered
3	Numbness	Transient numbness at the injection area	Nonserious	Recovered
	Pain	Transient pain at the lateral aspect of joint	Nonserious	Recovered
4	Fever	Transient fever up to 37.4℃ at POD-1	Nonserious	Recovered
	Stiffness	Transient stiffness at the joint	Nonserious	Recovered
5	Pain	Transient pain at the lateral aspect of joint	Nonserious	Recovered
	Dizziness	Transient dizziness at POD-2	Nonserious	Recovered

POD: post-operative day

**Table 4:** Clinical outcomes evaluation

	Pre-operative	Post-operative
VAS score	6.0(4.5,8.3)	3.5(2.0,5.0)
WOMAC score	26.0(21.0,37.0)	8.5(7.0,12.75)
SF-12 score	39.0(35.8,42.3)	46.0(44.0,48.3)
MOCART score	42.0(34.0,48.0)	58.0(49.0,68.3)

All data were presented as median (interquartile range). VAS: Visual Analog scale; WOMAC: Western Ontario and McMaster Universities index; SF-12: Short-form 12; MOCART: Magnetic Resonance Observation of Cartilage Repair Tissue

## Discussion

This study preliminarily confirmed that repeated UC-MSCs intra-articular injection for the treatment of knee osteoarthritis did not produce serious adverse reactions, providing a reference basis for further verification of the clinical application effect of this treatment. As a phase I clinical trial, we focused on treatment-related adverse events in patients. In this study, we observed the safety of intra-articular injection of UC-MSCs into the knee, and the results showed that some transient side effects, such as pain, fever, and stiffness, occurred after intra-articular injection, and these symptoms resolved spontaneously within a day or two, perhaps related to the relevant physiological responses of stem cells in the joint cavity. The remaining patients who did not experience these side effects reported feeling well after injection. This result suggests that the adverse effects are acceptable. Mechanism involved in swelling and pain after intra-articular injection of UC-MSCs is not very clear, which may be related to the induction of mild synovial inflammation by stem cells in the joint [21, 22]. Although UC-MSCs are of the same species origin, they also have the risk of triggering immune rejection as heterologous cells. In the previous studies, it has been shown that MSCs can promote the secretion of more GFs and CKs by tissues, which promotes the proliferation and differentiation of cells, while helping to regulate the inflammatory microenvironment and promote tissue regeneration [23, 24].

In addition to assessing the safety of treatment, we followed patients who received repeated intra-articular injection of stem cells and briefly analyzed the improvement of joint motion and pain after treatment. In this study, we used human UC-MSCs to clinically treat OA, and after treatment, the VAS score, WOMAC score, and SF-12 score of the patients were partially improved compared with those before treatment, which effectively improved the joint pain symptoms and joint movement of the patients, as well as the quality of life of the patients. The MRI results showed that the knee cartilage of the patients was repaired to some extent after receiving UC-MSCs treatment, indicating that stem cell therapy had a positive effect on cartilage repair. Orozco et al. [25] conducted a single-arm trial using autologous MSCs to investigate their therapeutic effect on OA. After one year of follow-up, knee pain and joint function were significantly improved in patients participated in the study. Vega et al. [26] also investigated cartilage repair effect using MSCs and found significant improvement in WOMAC scores, and radiographic MRI findings also showed significant improvement in cartilage morphology compared with pre-treatment. Park et al. [27] performed a clinical study of treating arthritis using allogeneic UC-MSCs and showed that there was a significant improvement in joint pain and function in patients. Their findings are similar to ours, which suggests that intra-articular injection of human umbilical cord mesenchymal stem cells may bring new research directions for treatment strategies for knee osteoarthritis.

At present, there is no uniform treatment specification or standard for UC-MSCs in the treatment of OA. In this study, all patients with OA received a total of four UC-MSCs injections, which is different from previous studies. UC-MSCs were injected only once or twice in previous studies, but the therapeutic effect for knee OA was not significant [28, 29]. After being injected into the joint cavity, UC-MSCs are directly exposed to an arthritic situation, and secrete substances such as GFs, CKs, and ligands that facilitate cartilage repair [30]. UC-MSCs may delay the destruction of cartilage tissue by inhibiting the expression of matrix-degrading enzymes in chondrocytes, and at the same time, it can promote the differentiation of cartilage progenitor cells and play a role in promoting cartilage repair. UC-MSCs injected into the joint can reduce the secretion of inflammatory factors to a certain extent, improve the microenvironment of chondrocyte growth, inhibit the inflammatory response of synovial tissue in the joint, and reduce the knee pain symptoms.

From the above mechanism, it can be concluded that stem cell concentration also has a crucial role for the treatment of OA. In this study, 1.5 × 10^7^ UC-MSCs were injected each time, which was slightly higher than those in previous similar studies [24]. After injected into the knee, the survival of stem cells is unknown, which may not allow the joint to maintain a higher concentration of stem cells for a long time. We chose to inject four times, which might increase the therapeutic effect. This may also induce the related adverse events, so we focused on observing the occurrence and outcome of AEs in patients. The results showed that the increase of stem cell concentration did not raise the probability of AEs in patients, and all AEs were transient and could resolve spontaneously, suggesting that the increase of stem cell concentration is riskless on the treatment of OA.

There were also some limitations to this study. Firstly, blinding was not used in selecting patients for inclusion in the study and there might have been selective bias. Secondly, this research is a phase I single-arm study, focusing on treatment-related adverse reactions, and the therapeutic effect has not been deeply explored. In addition, the number of patients was small and results were presented using descriptive data, and no statistical analysis was performed. Finally, the characteristic of patients in this study was limited, and other information, such as occupation, was not available, which might have affected the study results.

## Conclusions

In our study, we explored the AEs of repeated intra-articular injections of UC-MSCs in the treatment of OA, and found this therapeutic strategy was safe. Although some AEs emerged during the research, all of them were transient and did no harm to patient. In the future, large-scale multicenter randomized controlled trials can be carried out to investigate the efficacy of repeated injections of UC-MSCs in the treatment of knee OA.

## Data Availability

All data generated or analyzed during this study are included in this published article.
